# Supplementary material for: Computational modelling of the equine arteritis virus GP5/M Dimer: Implications for immune evasion and virulence
Source: PLoS One. 2026 Mar 10;21(3):e0344287. doi: 10.1371/journal.pone.0344287 (PMC12974795; doi:10.1371/journal.pone.0344287)
Supplement: S1 Fig — (PDF) [file pone.0344287.s001.pdf]

S1 figure

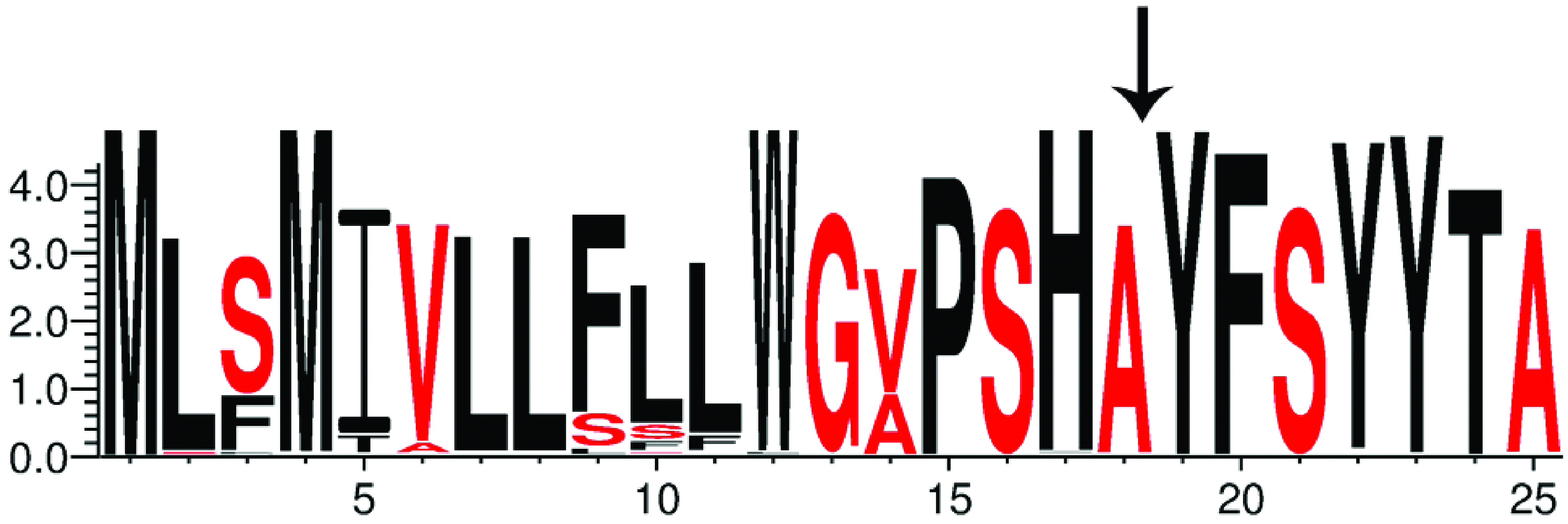

S1 figure: Web loge representation of the first 25 amino acids of the full-length Gp5 of EAV strains. The arrow indicates the only cleavage site predicted by SignalP 5. Small amino acids that determine signal peptide cleavage if present at the -1 and -3 position are shown in red.
