# Supplementary material for: Computational modelling of the equine arteritis virus GP5/M Dimer: Implications for immune evasion and virulence
Source: PLoS One. 2026 Mar 10;21(3):e0344287. doi: 10.1371/journal.pone.0344287 (PMC12974795; doi:10.1371/journal.pone.0344287)
Supplement: S2 Fig — (PDF) [file pone.0344287.s002.pdf]

S2 figure

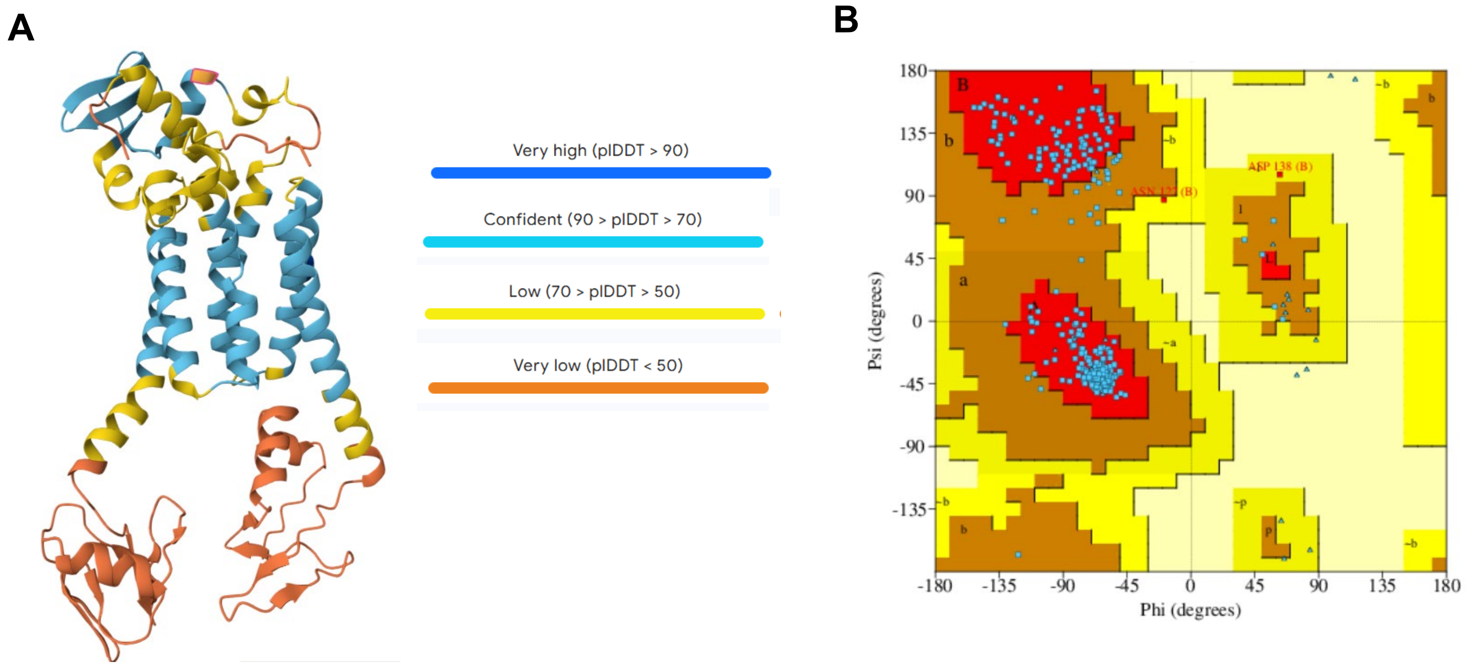

1. Ramachandran Plot statistics

		No. of residues	%-tage
Most favoured regions	[A,B,L]	320	91.2%
Additional allowed regions	[a,b,l,p]	29	8.3%
Generously allowed regions	[~a,~b,~l,~p]	2	0.6%
Disallowed regions	[XX]	0	0.0%
Non-glycine and non-proline residues		351	100.0%
End-residues (excl. Gly and Pro)		3	
Glycine residues		27	
Proline residues		18	
Total number of residues		399	

S2 figure: Quality scores for the Gp5/M model

A: Averaged per-residue pLDDT confidence score for individual protein domains

B. Ramachandran plot of the predicted structure of model 1. It shows the energetically allowed regions for backbone dihedral angles ψ against φ of amino acid residues in the Gp5/M structure. The φ-angel is the angel of the bond between the Cα-atom (that carries the amino acid side chain) to the N-atom and the ψ-angel is the angel between the Cα-atom and the other C-atom of the peptide. For steric reasons, only certain combinations of ψ and φ angels are allowed. The red, brown, dark yellow and light-yellow regions represent the favoured, allowed, “generously allowed” and unallowed regions, respectively. Only two amino acid Asn 122 and Asp138 of M) are in the generously allowed region and none in the unallowed regions.
