# Supplementary material for: Computational modelling of the equine arteritis virus GP5/M Dimer: Implications for immune evasion and virulence
Source: PLoS One. 2026 Mar 10;21(3):e0344287. doi: 10.1371/journal.pone.0344287 (PMC12974795; doi:10.1371/journal.pone.0344287)
Supplement: S3 Fig — (PDF) [file pone.0344287.s003.pdf]

## S3 figure

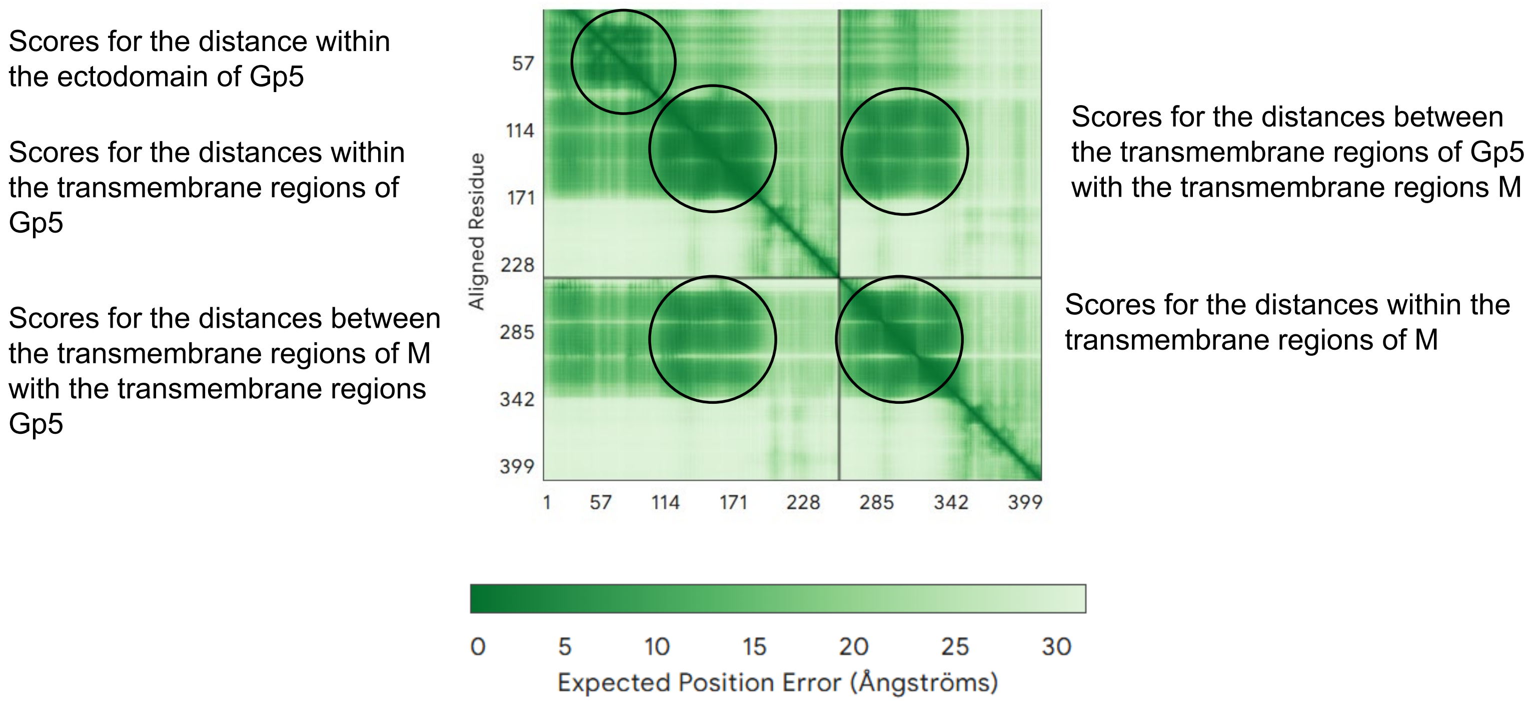


**S3 figure: Prediction aligned error (PAE) score of the predicted structure of Gp5/M of EAV Bucyrus.**
The prediction aligned error (PAE) measures confidence in the relative positions of pairs of amino acids. PAE is displayed as a 2D plot and the expected position error in Angstrom is color-coded. Both axes indicate the position of the individual amino acids.  The uncertainty in the predicted distance of two amino acids is color-coded from dark green (0Å) to light green(30Å), as shown in the lower bar. The colour of the intersection of a horizontal line drawn from the position of an amino acid on the y-axis and a vertical line from the position of another amino acid on the x-axis indicates the error in the predicted distance between these two residues. PAE graphs are always characterized by a diagonal green line, since amino acids that are juxtaposed in the primary sequence are also adjacent in the 3D structure. The upper, left quadrant corresponds to errors in the distances of residues within Gp5, the lower right quadrant to errors within M, the upper, right quadrant to errors between Gp5 and M, and the lower left quadrant to errors between M and Gp5.
