# Supplementary material for: Computational modelling of the equine arteritis virus GP5/M Dimer: Implications for immune evasion and virulence
Source: PLoS One. 2026 Mar 10;21(3):e0344287. doi: 10.1371/journal.pone.0344287 (PMC12974795; doi:10.1371/journal.pone.0344287)
Supplement: S4 Fig — (PDF) [file pone.0344287.s004.pdf]

S4 figure

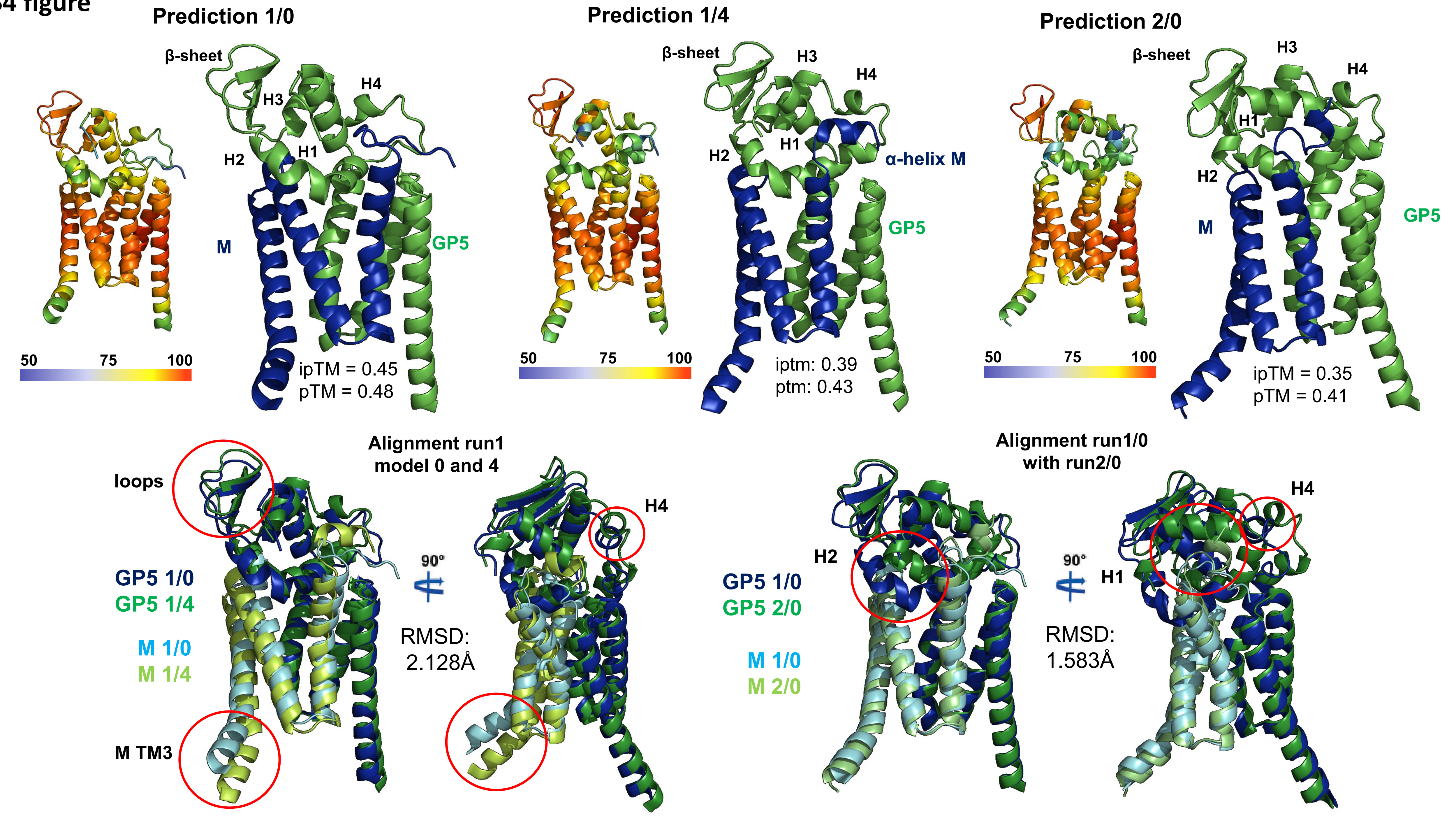
Prediction 1/0
β-sheet
H3
H4
H2
H1
M
GP5
50
75
100
ipTM = 0.45
pTM = 0.48
Prediction 1/4
β-sheet
H3
H4
H2
H1
α-helix M
GP5
50
75
100
iptm: 0.39
ptm: 0.43
Prediction 2/0
β-sheet
H3
H4
H1
H2
M
GP5
50
75
100
ipTM = 0.35
pTM = 0.41
Alignment run1
model 0 and 4
loops
GP5 1/0
GP5 1/4
M 1/0
M 1/4
M TM3
90°
RMSD:
2.128Å
H4
Alignment run1/0
with run2/0
H2
GP5 1/0
GP5 2/0
M 1/0
M 2/0
90°
RMSD:
1.583Å
H1
H4

S4 Figure. Comparison of different AlphaFold 3 models of GP5/M of EAV. Models were generated in two independent runs, each producing five structures ranked from 0 to 4 according to their pTM and iPTM scores, which estimate the accuracy of the predicted global fold (pTM) and the relative orientation of protein subunits or domains (iPTM).Upper panel: Per-residue confidence (pLDDT) shown using a rainbow gradient from red (high confidence) to blue (low confidence), together with a cartoon representation of the EAV GP5/M dimer (GP5 in green, M in blue). Lower panel: Superposition of selected AlphaFold 3 models. Left: alignment of models 0 and 4 from run 1 (1/0 and 1/4). Right: alignment of model 0 from run 1 with model 0 from run 2. Deviations in the spatial positioning of the protein chains between models are highlighted with red circles. pTM estimates the overall accuracy of the predicted fold, while ipTM specifically measures the confidence in the inter-chain interface of a protein complex.
