# Supplementary material for: Computational modelling of the equine arteritis virus GP5/M Dimer: Implications for immune evasion and virulence
Source: PLoS One. 2026 Mar 10;21(3):e0344287. doi: 10.1371/journal.pone.0344287 (PMC12974795; doi:10.1371/journal.pone.0344287)
Supplement: S5 Fig — (PDF) [file pone.0344287.s005.pdf]

S5 figure

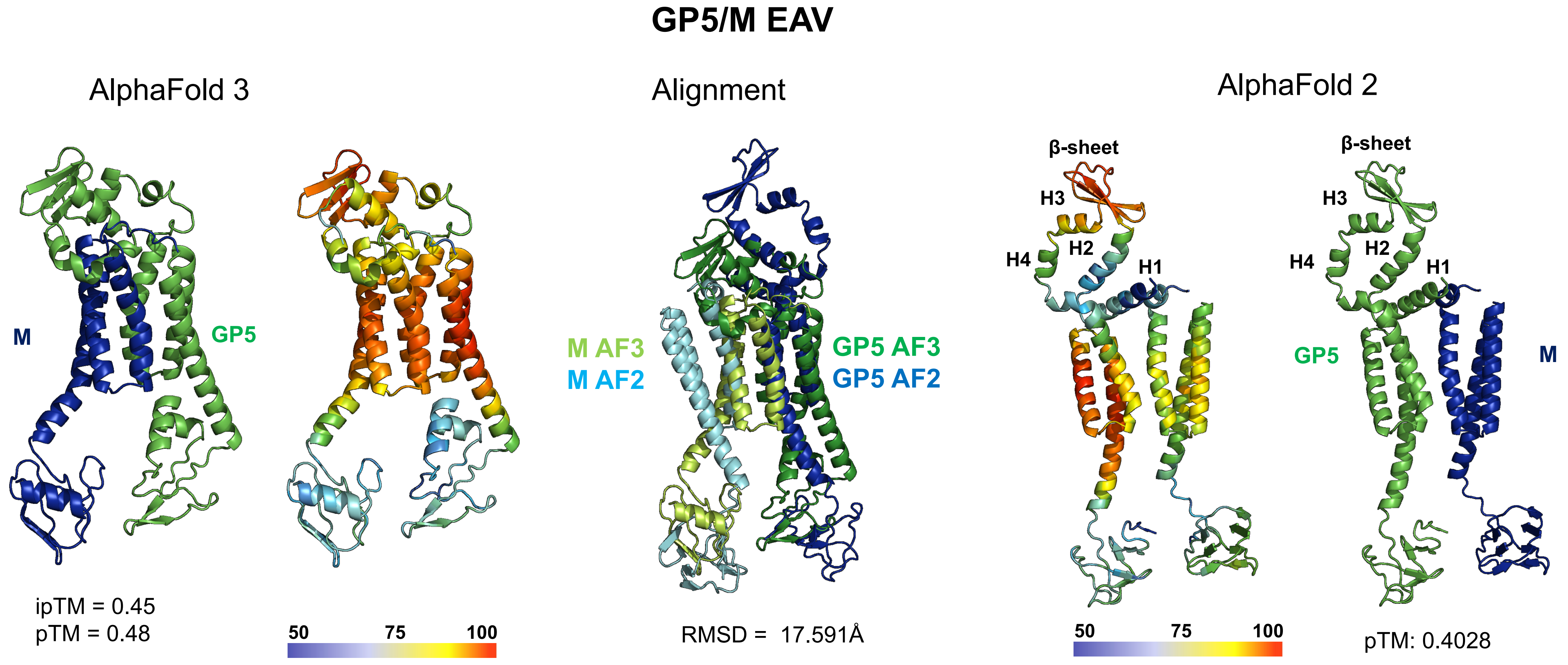

S5 Figure. Comparison of the AlphaFold 2 and AlphaFold 3 models of the EAV GP5/M dimer.
Left and right panels: Per-residue confidence (pLDDT) shown using a rainbow gradient from red (high confidence) to blue (low confidence), together with cartoon representations of the GP5/M dimer. Middle: Structural alignment of the AlphaFold 2 and AlphaFold 3 models. The superposition yields an RMSD of approximately 17 Å, indicating that no meaningful alignment between the two models is possible. This large deviation, along with the better pLDDT scores of the AlphaFold 3 model, demonstrates a substantial improvement in the EAV GP5/M dimer prediction. pTM estimates the overall accuracy of the predicted fold, while ipTM specifically measures the confidence in the inter-chain interface of a protein complex.
