# Supplementary material for: Computational modelling of the equine arteritis virus GP5/M Dimer: Implications for immune evasion and virulence
Source: PLoS One. 2026 Mar 10;21(3):e0344287. doi: 10.1371/journal.pone.0344287 (PMC12974795; doi:10.1371/journal.pone.0344287)
Supplement: S6 Fig — (PDF) [file pone.0344287.s006.pdf]

S6 figure

GP5/M PRRSV-2 VR 2332

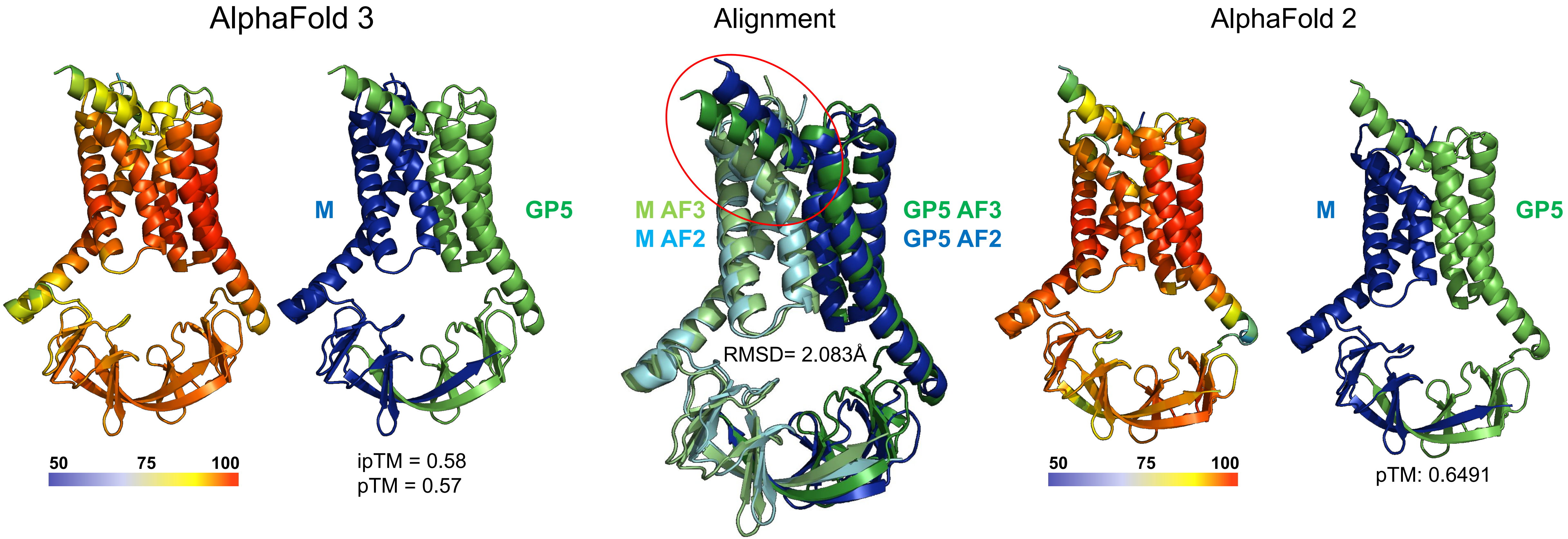

S6 Figure. Comparison of the AlphaFold 2 and AlphaFold 3 models of the PRRSV-2 GP5/M dimer.
Left and right panels: Per-residue confidence (pLDDT) shown using a rainbow gradient from red (high confidence) to blue (low confidence), together with cartoon representations of the GP5/M dimer. Middle: Structural alignment of the AlphaFold 2 and AlphaFold 3 models. The superposition yields an RMSD of approximately 2 Å, indicating a close structural match; the only notable difference is the spatial position of the N-terminal helix of GP5, highlighted by the red circle. In contrast to EAV, this small deviation, together with the largely unchanged pLDDT scores, shows that AlphaFold 3 does not substantially improve the GP5/M model for PRRSV-2. pTM estimates the overall accuracy of the predicted fold, while ipTM specifically measures the confidence in the inter-chain interface of a protein complex.
