# Supplementary material for: Computational modelling of the equine arteritis virus GP5/M Dimer: Implications for immune evasion and virulence
Source: PLoS One. 2026 Mar 10;21(3):e0344287. doi: 10.1371/journal.pone.0344287 (PMC12974795; doi:10.1371/journal.pone.0344287)
Supplement: S7 Fig — (PDF) [file pone.0344287.s007.pdf]

S7 figure

```
Multiple sequence alignment with Clustal_Omega
EAV         -YFSYYTAQRFTDFTLCMLTDRGVIANLLRYDEHTALYNCSASKTCWYCTFLDEQIITFG    59
PRRSV1      -NGDSSTYQYIYNLTICELNGTD-------------------------------------    22
PRRSV2      SNDSSSHLQLIYNLTLCELNGTD-------------------------------------    23
               .    * : ::*:* *.. .

EAV         TDCDDTYAVPVAEVLEQAHGPYSALFDDMPPFIYYG 95
PRRSV1      ------------------------------------ 22
PRRSV2      ------------------------------------ 23
```

S7 figure: Multiple sequence alignment of the ectodomains of Gp5 of EAV, PRRSV-1 and PRRSV-2
The alignment was done using ClustalOmega https://www.ebi.ac.uk/jdispatcher/msa/clustalo?stype=protein using the amino acid sequences of the ectodomains without the signal peptide from EAV, strain Bucyrus, PRRSV-1 reference strain Lelystad and PRRSV-2 reference strain VR 2332. (asterisk) * indicate conserved, (colon): strongly similar and (period) . weakly similar residues
