# Supplementary material for: Computational modelling of the equine arteritis virus GP5/M Dimer: Implications for immune evasion and virulence
Source: PLoS One. 2026 Mar 10;21(3):e0344287. doi: 10.1371/journal.pone.0344287 (PMC12974795; doi:10.1371/journal.pone.0344287)
Supplement: S8 Fig — (PDF) [file pone.0344287.s008.pdf]

S8 figure

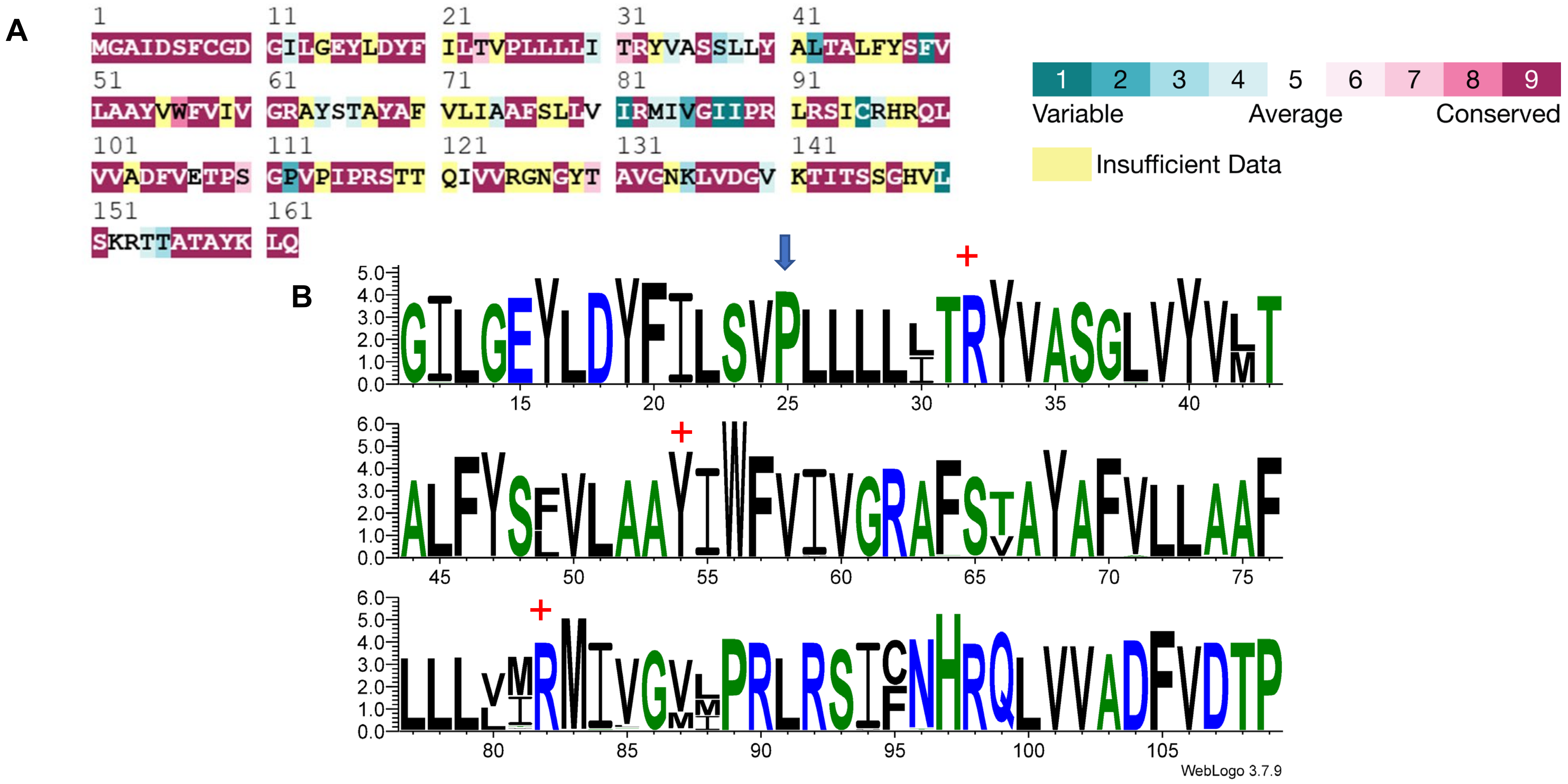

S8 Fig. Conservation of amino acids in M

(A) Evolutionary conservation scores calculated using ConSurf are mapped onto the amino acid sequence of M. The color scale ranges from variable (turquoise) to highly conserved (maroon); residues lacking sufficient data are shown in yellow as highlighted in the bar.

(B) Web logo showing the amino acids at each position in the transmembrane region of M. The overall height of the stack indicates the sequence conservation at a position (X-axis), while the height of symbols within the stack indicates the relative frequencry of each amino. Proline causing a kink in TM1 is indicated by an arrow and esidues predicted to interact with the transmembrane region of Gp5 are denoted indicated by a plus sign (+).
