# Supplementary material for: Computational modelling of the equine arteritis virus GP5/M Dimer: Implications for immune evasion and virulence
Source: PLoS One. 2026 Mar 10;21(3):e0344287. doi: 10.1371/journal.pone.0344287 (PMC12974795; doi:10.1371/journal.pone.0344287)
Supplement: S10 Fig — (PDF) [file pone.0344287.s010.pdf]

S10 figure

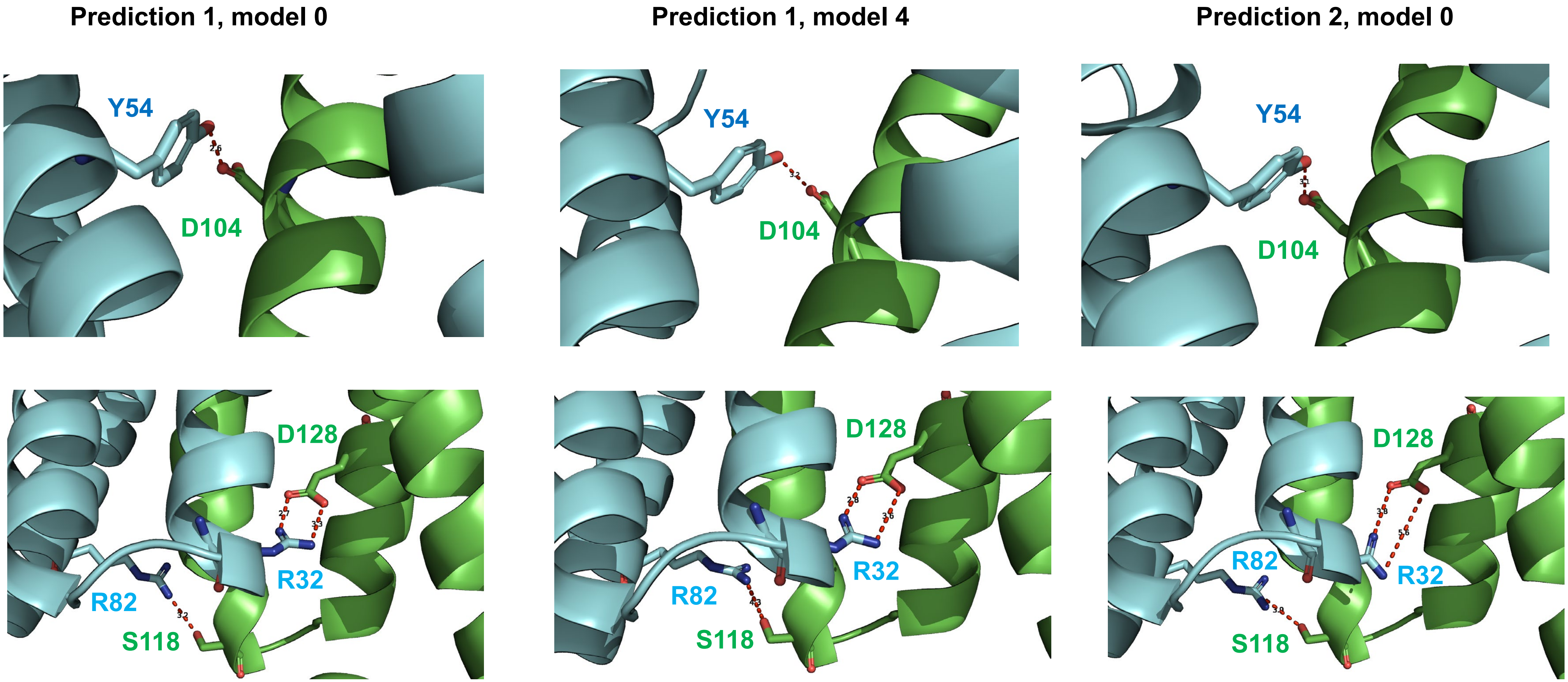

S10 figure: Details of parts of the transmembrane regions of three alphafold3 models of GP5/M of EAV. Identical electrostatic interactions between the transmembrane regions of GP5 and M were observed across all three models. Interaction sites are shown as sticks and labeled.
