# Supplementary material for: Computational modelling of the equine arteritis virus GP5/M Dimer: Implications for immune evasion and virulence
Source: PLoS One. 2026 Mar 10;21(3):e0344287. doi: 10.1371/journal.pone.0344287 (PMC12974795; doi:10.1371/journal.pone.0344287)
Supplement: S11 Fig — (PDF) [file pone.0344287.s011.pdf]

S11 figure

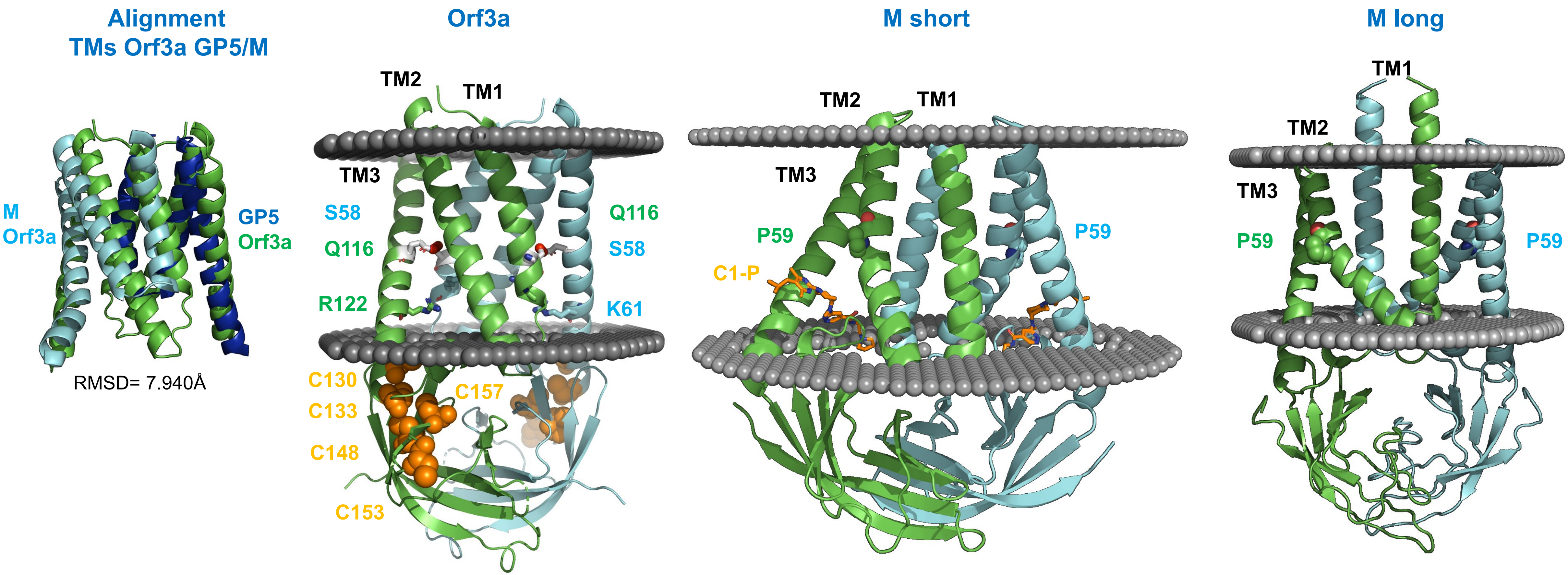

S11 Fig. Structure of Orf3a and M of SARS-CoV-2 in a membrane context

Structure of Orf3a (PDB: 6XDC) and of two conformational states of the M protein. Short conformation (M short, PDB: 9CTU) showing a compact dimer with Golgi-enriched sphingolipid ceramide-1-phosphate (C1-P, orange spheres) binding site. C1-P specifically binds to the short form, thereby stabilizing this conformation which recruits the nucleocapsid to the budding site. In the long conformation (M long, PDB: 7VGR), the dimer adopts an elongated interface that promotes M protein oligomerization, driving virus budding. Left part: Alignment of the transmembrane regions of ORF3a with GP5/M of EAV. RMSD: Root-Mean-Square Deviation. No meaningful alignment of the GP5/M transmembrane regions with either the long or short form of M was achievable.
