# Supplementary material for: Computational modelling of the equine arteritis virus GP5/M Dimer: Implications for immune evasion and virulence
Source: PLoS One. 2026 Mar 10;21(3):e0344287. doi: 10.1371/journal.pone.0344287 (PMC12974795; doi:10.1371/journal.pone.0344287)
Supplement: S12 Fig — (PDF) [file pone.0344287.s012.pdf]

S12 figure

	Gp5 VR	Gp5 Lely	Gp5 EAV	M VR	M Lely	M EAV	Orf3a SARS	M SARS
**Gp5 VR**	**X**	62	19	10	12	16	15	15
**Gp5 Lely**	61 / 72	**X**	21	11	11	15	16	15
**Gp5 EAV**	17 / 30	17 / 26	**X**	14	12	16	11	16
**M VR**	18 / 33	18 / 28	17 / 27	**X**	78	25	13	18
**M Lely**	19 / 30	18 / 31	21 / 30	79 / 91	**X**	27	14	20
**M EAV**	16 /26	22 / 31	14 / 24	24 / 37	26 / 40	**X**	17	20
**Orf3a SARS**	4 / 6	10 / 17	12 / 19	15 / 21	11 / 21	12 / 24	**X**	12
**M SARS**	15 / 25	20 / 29	13 / 23	16 / 28	15 / 30	18 / 30	14 / 23	**X**

S12 figure: Amino acid sequence conservation between Gp5 and M of Arteriviruses and Orf3a and M of SARS-CoV-2. The numbers above the diagonal where generated by a multiple sequence alignment of all amino acid sequences using Clustal Q https://www.ebi.ac.uk/Tools/msa/clustalo/ and are the calculated identity matrix.
The numbers below the diagonal were generated by a pairwise sequence alignment using needle https://www.ebi.ac.uk/Tools/psa/emboss_needle/ and are the percentage of identical and similar amino acids, respectively for each pairwise alignment. The predicted signal peptides of Gp5 were removed from the sequence prior to both analysis. Highlighted in blue: sequence similarity between Gp5 of EAV with the other proteins. Highlighted in grey: sequence similarity between M of EAV with the other proteins. Highlighted in yellow: sequence similarity between M and Gp5 of EAV.
