# Supplementary material for: Computational modelling of the equine arteritis virus GP5/M Dimer: Implications for immune evasion and virulence
Source: PLoS One. 2026 Mar 10;21(3):e0344287. doi: 10.1371/journal.pone.0344287 (PMC12974795; doi:10.1371/journal.pone.0344287)
Supplement: Attachment — Submitted filename: point by point reply.pdf [file pone.0344287.s015.pdf]

Point-by-point reply

Reviewer #1: This is a review on the manuscript entitled "Computational Modelling of the Equine Arteritis Virus GP5/M Dimer: Structural Basis for Immune Evasion, Virulence and Virus Budding" by Michael Veit and Anna Karolina Matczuk. Equine arteritis virus (EAV) is an important viral pathogen in horses. The major viral envelop proteins GP5 and M form heterodimer that is critical for virus assembly and budding. These proteins contain immunogenic epitopes that involve in viral pathogenesis and host immune responses. In this study, the authors used the AI-driven tool AlphaFold3 to predict the 3D structure of the EAV GP5/M dimer and compared to its homolog in porcine reproductive and respiratory syndrome virus (PRRSV) and SARS-CoV-2. Their findings provide in-depth molecular insights into the structure-function of the GP5/M dimer and established a foundation for rational design of EAV vaccines. This is a well-written manuscript with detailed protein structure analysis. This reviewer only has a few of the following comments/suggestions for the authors to address.

1. Line 41-43, "Other relevant arteriviruses include lactate dehydrogenase–elevating virus (LDV) and simian hemorrhagic fever virus (SHFV), the latter contains viruses with zoonotic potential": The arterivirus family has been expanded and reclassified into 6 subfamilies containing 23 species with a number of more recently identified members, many of which are originated from monkeys. This information needs to be updated with references in the manuscript.

The information was updated:

Line 38-40: "***Alphaarterivirus equid*, common name** equine arteritis virus (EAV), which causes respiratory and reproductive disease in horses **and donkeys**, is an enveloped plus-strand RNA virus in the Arteriviridae family, **which comprises 6 subfamilies."**

Line 44-46: "Other relevant arteriviruses include lactate dehydrogenase–elevating virus (LDV) **and many species of subfamily Simarterivirinae including** simian hemorrhagic fever virus (SHFV), **which infect non-humane primates and possess** zoonotic potential (2-5)."

2. Line 152-153, "Only one GP5 sequence is predicted to be cleaved between residues 16 and 17…..": More information is needed for this specific GP5 sequence. Is there a deletion in GP5 sequence or a possible annotation/sequencing error?

We have added the following information to the legend of Figure S1 rather than to the main text, in order to avoid overloading the manuscript with technical detail:

"The arrow marks the signal peptide cleavage site predicted by SignalP 5.0, located between residues 18 and 19 in all GP5 sequences except one. Amino acids at the −1 and −3 positions

that promote signal peptide cleavage are highlighted in red. **In the exceptional GP5 variant (NCBI Protein accession ABL14296.1), the small residue Ala at the −1 position is replaced by the bulky residue Asp, which prevents cleavage at this canonical site. Instead, SignalP predicts an alternative cleavage between residues 16 and 17 for this strain, accompanied by a marked reduction in the overall cleavage probability (from ~0.9 to ~0.4)**."

3. Lines 185-188: The rainbow pLDDT figure is helpful. It will be better if the authors describe more precisely which transmembrane region and β-sheet exhibit the highest confidence.

We added the following information to this sentence (lines 193-195):

"This representation highlights that the membrane spanning parts of all six transmembrane regions and a prominent β-sheet and the following helix 1 within the GP5 ectodomain are predicted with the highest confidence, underscoring their structural reliability (Fig 1A, B, 2A)."

4. Lines 248–249: The authors noted that the EAV GP5 ectodomain (89 aa) is longer than PRRSV GP5 (26 aa). It is better to further discuss the functional implications of this extended domain and how it may relate to biological differences between EAV and PRRSV.

We added the following sentence to this part of the manuscript (lines 293-295): "The extended GP5 ectodomain of EAV may influence receptor engagement and other host-factor interactions, which aligns with the broader cell and tissue tropism of EAV."

5. Lines 394–396: Part of this paragraph should be moved to discussion section. The structural analysis suggests that protein surface/epitope exposure could influence immune recognition. It is better to provide a discussion of previous studies in cell culture or animal models to support this notion.

We have removed the final sentence of this paragraph: "This structural rearrangement likely repositions amino acids locally, potentially exposing new residues on the molecule's surface.". We think that a more extensive treatment of its immunological implications would go beyond the scope of the present manuscript

6. Line 460, ".... with orf3a (12/19": This sentence does not seem to be completed. It should be written as ".....with SARS-CoV-2 orf3a (12%/19%)."

The sentence was corrected accordingly.

Reviewer #2: Dear authors, the work is extremely interesting, and you have achieved a very good result using computer programs. Regarding figure 4, point C, I suggest highlighting the epitopes denoted in orange, blue, black, and green, which are not clearly visible, so that they can be properly appreciated.

We thank the referee very much for the positive and encouraging comment. In the revised figure 4c, we have now labelled the four epitopes in the protein sequence with the letters A–D so that they clearly indicate the color-coded epitopes shown in the structural representation.

Reviewer #3: Comments to the Authors

Title: The title implies experimentally validated mechanisms ("structural basis for virus budding") that are not demonstrated. A more conservative title reflecting the predictive nature

We agree with this concern and deleted "virus budding" from the title and also replaced "structural basis" with "implications: "Computational Modelling of the Equine Arteritis Virus GP5/M Dimer: Implications for Immune Evasion and Virulence ~~and Virus Budding~~"

Introduction: While the study focuses on EAV, the Introduction places disproportionate emphasis on PRRSV GP5/M biology. This weakens the narrative focus and makes EAV appear primarily as a comparative extension. The Introduction should more clearly define the specific knowledge gap for EAV and justify the comparative framework.

We thank the referee for this thoughtful comment. We carefully reviewed the Introduction in light of this suggestion. While we appreciate the concern about balance, we respectfully note that the Introduction is structured primarily around EAV. After the general overview of the Arteriviridae family (lines 38–49), the subsequent sections on pathogenesis (lines 50–57), vaccines (lines 58–66), viral receptors (lines 81–92), and antibody epitopes (lines 118–123) focus exclusively on EAV. The section on GP5/M processing (lines 98–117) includes selected PRRSV studies because most mechanistic insights available in the literature stem from PRRSV—particularly PRRSV-2 strain VR-2332—and these basic processes are considered highly conserved between PRRSV and EAV. To ensure completeness, we added an additional EAV-specific reference and now cite all published studies on GP5/M processing in EAV that we are aware of.

The specific knowledge gap addressed in this study is defined later in the Introduction. In line 123, we state: "Despite its critical role in virus replication and immune evasion, the three-dimensional structure of the GP5/M complex remains unknown."

Furthermore, lines 140–142 outline the aim of the study: "In this study, we employed AlphaFold3 to predict the GP5/M structure of EAV, focusing on known antibody epitopes and virulence factors. Knowing the precise 3D structure of an antigenic protein enables researchers to identify surface-exposed epitopes."

Interpretation of Structural and Evolutionary Comparisons: The reported structural similarities between arterivirus GP5/M and SARS-CoV-2 M/ORF3a are interesting, but conclusions should

be limited to conserved topology and fold. Functional or evolutionary equivalence cannot be inferred from AlphaFold3 predictions alone, particularly given their static nature.

AND

Limitations: The study relies exclusively on computational modeling. Regions with low confidence, especially the endodomains, limit interpretation of cytoplasmic interactions. Claims related to virus budding and assembly should therefore be explicitly presented as speculative and hypothesis-generating.

Thank you for these valuable comments. We have revised and condensed the summary at the end of the discussion to clearly acknowledge the limitations of an exclusively computational approach, including the low-confidence regions in the cytoplasmic endodomains. The text now explicitly states that AlphaFold3 predictions cannot support functional, mechanistic, or evolutionary equivalence and that any interpretations related to cytoplasmic interactions, budding, or assembly are speculative. (lines 632-644).

"In summary, this study demonstrates that the AlphaFold3-predicted structure of the EAV GP5/M dimer reliably captures the architecture of parts of the ectodomain and transmembrane regions, offering new molecular insights into its organization. The model enables precise mapping of virulence-associated determinants and known antibody epitopes, which is consistent with a dual immune evasion strategy that combines antigenic drift with glycan shielding. At the same time, AlphaFold3 predictions reflect conserved topology and overall fold but cannot establish functional or evolutionary relationships. Because the models are computational and static, and several regions, particularly the cytoplasmic endodomains, are predicted with low confidence, functional or mechanistic equivalence cannot be inferred. Within these limitations, the conserved architecture of the transmembrane domains of Arterivirus GP5/M and those of SARS-CoV-2 M and ORF3a is consistent with, but does not demonstrate, the possibility of a distant shared ancestry or common principles of membrane-associated assembly within the Nidovirales."

Reviewer #4: Dear authors, the work is extremely interesting, and you have achieved a very good result using computer programs. Regarding figure 4, point C, I suggest highlighting the epitopes denoted in orange, blue, black, and green, which are not clearly visible, so that they can be properly appreciated.

We thank the referee very much for the positive and encouraging comment. In the revised figure 4c, we have now labelled the four epitopes above the protein sequence with the letters A–D so that they clearly indicate the color-coded epitopes shown in the structural representation.

Reviewer #5: This manuscript uses AlphaFold3 to model the EAV GP5/M heterodimer, compares it with PRRSV homologs, and maps neutralizing epitopes, N-glycosylation sites, and virulence/persistence mutations to propose mechanisms of immune evasion and morphogenesis. The topic is relevant and the structure-guided mapping is potentially useful, especially the discussion linking epitope variability with nearby glycans and membrane-proximal mutations with functional phenotypes.

The main weakness is model confidence: the reported best model has pTM=0.48 and low-confidence endodomain regions (pLDDT<50), so several long-range interpretations (subunit arrangement, glycan "shielding," and broad cross-Nidovirales extrapolations) need stronger quantitative support and more conservative wording.

1. Lines 173-175: The manuscript should more explicitly bound which conclusions are supported by high-confidence regions versus low-confidence regions, given the reported pTM=0.48 and low pLDDT in the endodomain; the authors should test robustness across the five AF3 candidate models by reporting whether key features (apical epitope region, helix4 membrane-proximal region, and the proposed interface) are consistent across models (e.g., segment RMSD and interface-contact overlap), rather than basing interpretation on a single "best" model.

We thank the reviewer for raising this important point. In response, we performed an additional AlphaFold3 prediction and compared two models from the initial run (the highest- and lowest-confidence models) with one independently generated model from a second run. AlphaFold3 generates each model by iteratively denoising a cloud of random initial atomic positions, with each model initialized by a unique Seed. This allows us to assess the robustness of structural features across independently seeded predictions.

We have added the following text to the manuscript (lines 231-251): "We then compared the models with the highest and lowest confidence scores and performed an additional AlphaFold3 run to ensure convergence. Unlike deterministic calculations, AlphaFold3 builds structures by iteratively 'denoising' a cloud of random initial atomic positions. This process is governed by a 'Seed'—a starting number for the random number generator. Since each model, whether from the same or different runs, is initialized with a unique Seed, the algorithm explores a slightly different pathway during structure construction. This ensemble approach allows to further assess the reliability of the model. Consistent results across seeds indicate a robust, well-defined structure, while variability highlights regions of either physical flexibility or uncertainty in the prediction.

All three models exhibit the same set of structural elements in the same order, with the only notable exception being the M-protein N-terminus, which forms a short α-helix in one model but remains unstructured in others. Mapping of pLDDT scores confirms that the six

transmembrane regions, the β-sheet, and helix 3 of the GP5 ectodomain are predicted with high confidence and align closely across all models. A minor misalignment is present in helix 4 across all models. A more pronounced deviation is observed between helices 1 and 2 when comparing the models from the two runs. Despite these localized variations in flexible loops and the N-terminus, the high structural convergence and confidence scores across the transmembrane core and key ectodomain elements demonstrate that the predicted heterodimer is robust."

We also expanded the Results section to clarify which conclusions are supported by high-confidence regions and to indicate where the models show consistent structural features. After the section on epitope localization, we added (lines 338-340): "Note that the β-sheet and the following helix 3 are predicted in all models with the highest accuracy and their structures align well."
After the section on virulence determinants, we added (lines 446-448): "Note that the spatial position of helix 4 varies among the AlphaFold models, but it consistently remains connected to helix 3 by a loop, and helix 3 itself aligns very well across all models (S4 Fig.)."
Finally, in the paragraph describing the transmembrane regions, we added (lines 483-485): "Interestingly, all three models predict identical electrostatic interactions between the transmembrane segments of GP5 and M, indicating that the prediction of the interface is robust (S10 Fig)."

2. The Methods should specify AF3 server/version/date, whether MSA/templates were used, exact sequence boundaries (signal peptide removal and any truncations), and the criteria for selecting the final model beyond global pTM; for a membrane-protein heterodimer, interface confidence is central, so the authors should report interface-relevant confidence (interface PAE or per-residue confidence at interface) and provide the final PDBs and basic visualization/analysis steps as supplementary materials.
This part of the Methods section (line 647) was substantially expanded and now contains the requested information.

3. Lines 500-505: As mapping epitope B/C variability near N38/N63 and the outbreak-associated N55 glycosylation, the authors should add quantitative exposure metrics (SASA of epitope residues, and occlusion estimates after adding simplified glycans), and either include a formal selection analysis (dN/dS/site tests) or soften "positive selection" language to "consistent with immune pressure," since variability alone is not decisive.
We thank the referee for this suggestion. We agree that quantifying the degree of glycan shielding would ideally strengthen our hypothesis regarding immune evasion via antigenic drift

and glycan masking. However, we believe that providing specific SASA and occlusion values for this AlphaFold3-derived model may be methodologically premature and potentially misleading for the following reasons:

Incompatibility with Automated Glycoprotein Builders: We attempted to model the glycans using standard tools (such as GLYCAM-Web). However, these tools failed to recognize N55 and N63 as viable glycosylation sites within the predicted local geometry of the AF3 model, despite these being experimentally verified sites. Furthermore, automated placement at N38 resulted in stereochemical clashes with the predicted membrane-proximal regions, orienting the glycan toward the viral membrane rather than the solvent. This suggests that the AF3-predicted orientation of these specific side chains may not be optimized for post-translational modification modeling.

Limitations of Static Modeling for Glycan Shielding: AlphaFold3 predicts the protein in an aglycosylated, static state. In reality, N-linked glycans are highly flexible and exist as a dynamic "ensemble" or "cloud" of conformations. Calculating a single SASA value based on a static, manually placed "simplified" glycan does not accurately represent the biological shielding effect, which is a function of the glycan's excluded volume over time. We are concerned that reporting a specific "percentage of occlusion" would imply a level of precision that a predicted static model cannot currently support.

However, to address the referee's concern regarding the limitations of our model, we have adjusted the language in our conclusion to emphasize its speculative nature. We changed the final sentence of the paragraph to the following (lines 561-562): "This elevated variability ~~indicates that both sites are likely subject to~~ is consistent with positive selection driven by antibody-mediated immune pressure."

4. Lines 383-396: In Fig 5, for virulence/persistence-associated mutations clustered near helix4 of Pro80/Ser82/Asp86 and related sites, the structural interpretation should include alternative mechanisms (notably indirect effects via glycosylation changes such as loss of the N63 site) and propose concrete validation experiments. GP5/M interaction assays with GP2/3/4, VLP budding/assembly assays, or targeted interface mutagenesis, to support the "interaction surface" hypothesis rather than presenting it as a primary explanation.

We thank the reviewer for this thoughtful comment. Because our structural interpretation in this section was speculative, we have removed the final sentence ("This structural rearrangement likely repositions amino acids locally, potentially exposing new residues on the molecule's surface."). With this speculative functional explanation removed, we consider it more appropriate not to propose specific experimental approaches in this manuscript, which focuses on structural prediction rather than functional validation.

Reviewer #6: The paper presented by Veit and Matczuk is an extension of their previous in silico work performed on the PRRSV GP5/M heterodimer, by predicting the structure of the EAV GP5/M heterodimer and comparing its predicted structural features to PRRSV and coronavirus proteins. Whilst the work allows to speculate about structural conservation and the importance of certain domains as neutralising epitopes, no in vitro experimental evidence is provided to strengthen the findings, making it difficult to assess the relevance of the presented data. Therefore, this study is a nice piece of in silico modelling and prediction, which can provide the interested scientific community with structure prediction based ideas to improve our understanding of EAV biology and vaccine design.

Major comments:

Please be carefully with the phrasing you are using. You are not working with an experimentally determined structure, but with a structural model. Hence, I would kindly ask you to clearly indicate that you are working with an in silico generated model in the whole manuscript, including the title., e.g. 'Computational modelling of the EAV GP5/M dimer suggests structural mechanisms of …'

We changed the title to the following:

"Computational Modelling of the Equine Arteritis Virus GP5/M Dimer: Implications for Immune Evasion and Virulence ~~and Virus Budding"~~

Did you also recalculate the structure of the PRRSV GP5/M dimer using AlphaFold3 to assess the structural similarity between AlphaFold2 and 3 predictions? I think this would be an important and interesting addition, or it should at least be discussed why this was not done.

We recalculated the structure of the PRRSV GP5/M dimer using AlphaFold3. However, in contrast to the EAV GP5/M dimer, this did not result in any notable improvement in model quality. The result is now shown in S5 and S6 Fig and described in the text (lines 269-279):

"When using AlphaFold3 instead of AlphaFold2, we observed a substantial improvement in the predicted structure of the EAV GP5/M dimer. In the AlphaFold2 model, only the GP5 β-sheet and the M-protein transmembrane helices TM2 and TM3 were predicted with moderate confidence, and no interactions between GP5 and M, including the conserved disulfide bond, were recovered. In addition, no meaningful structural alignment between the AlphaFold2 models was possible (S5 Fig). To evaluate whether similar improvements occur for PRRSV, we also recalculated the GP5/M dimer using AlphaFold3. In contrast to the EAV GP5/M model, the overall quality scores were comparable to the AlphaFold2 prediction, although regions with low to moderate confidence were located in different parts of the protein. Structural alignment of the two models yielded an RMSD of ~2 Å, with the only notable difference occurring in the helical N-terminus of GP5 (S6 Fig).

Figures in general:

Please adept the colours in the figures used to distinguish the M and the GP5 chain. They are too similar for easy distinction (e.g. green and pale blue).

We changed the colour from cyan to blue in Fig. 1 in order to make it better distinguishable from green.

Please add a scale of the color gradient used whenever you depict the pLDDT. Please also consider to use the 'usually' used Alphafold output colour code, which is blue for high confidence, and red for low confidence.

We have now added a scale bar indicating the pLDDT color gradient to every figure in which pLDDT values are shown.

Minor points:

Line 37 and 47 are repetitive. I think it would be important to indicate somewhere in the abstract that GP5/M are however not relevant for PRRSV neutralisation.

Line 37 has been rephrased: Alphaarterivirus equid, common name equine arteritis virus (EAV), which causes respiratory and reproductive disease in horses and donkeys, is an enveloped plus-strand RNA virus in the Arteriviridae family, which comprises 6 subfamilies" and line 47 was deleted.

The role of GP5/M in PRRSV neutralization remains incompletely resolved in the current literature, as also noted in the most recent review (40). Nevertheless, to highlight the distinction between PRRSV and EAV, we have added the following sentence to the introduction (lines 119-120): "This contrasts with PRRSV, in which the GP2/3/4 complex also serves as a target of neutralizing antibodies (40)."

Line 48: Please give references and numbers for the significant economic losses.

The sentence (lines 52-54) was changed to: "Economic losses in the equine industry arise from the multifaceted impact of EAV on reproduction, young horses and breeding operations and cost associated with surveillance, testing and animal movement and trade, which are difficult to estimate (10)."

Line 62: This reviewer is not sure such a statement is correct given which information is currently available in the literature, the statement should be weakened…

We are not sure whether the referee refers to line 62 or line 61 and therefore we modified both sentences.

Line 62 states: "Arteriviruses are composed of a helical nucleocapsid, comprising the viral RNA wrapped by the nucleocapsid protein N." The only potentially contentious aspect of this

sentence concerns the organization of the nucleocapsid. Earlier schematic representations of arterivirus particles depicted the capsid as icosahedral, but Cryo-EM analysis (Ref. 17) has demonstrated that the nucleocapsid instead adopts an asymmetric, linear arrangement rather than an isometric core. To reflect these structural data more precisely, we have revised the sentence (line 67) to: „Arteriviruses are composed of a ~~helical~~ nucleocapsid **with an asymmetric, linear organization**, comprising the viral RNA wrapped by the nucleocapsid protein N,… "

Line 61 states: "Although stallions are routinely screened prior to breeding and PCR testing is commonly performed before equine competitions, outbreaks of EAV continue to occur. but are probably underreported"

We agree that the available literature provides limited and sometimes outdated information on recent EAV outbreaks. From our experience, EAV remains underreported despite being a notifiable disease in the EU. For example, the most recent outbreak we are aware of occurred in Poland in 2019 (Wielkopolska Voivodeship), although no samples were accessible for analysis. According to expert communication from Prof. Jerzy Rola (Polish National Reference Laboratory, Puławy), approximately 10% of sera submitted for seroneutralization testing in 2024–2025 were EAV-positive and the vaccine was not available for many years.

To reflect this unpublished observation, we added the phrase 'but are probably underreported' to the sentence (line 66) and included a new reference to a documented EAV outbreak in the UK in 2019 (reference 12).

Line 149-151: Please indicate the tool used.

We have now specified the tool used by adding "using SignalP 5.0" to line 156. SignalP 5.0 is described in detail in the Methods section.

Line 156: What is 'web logo'? E.g. please introduce the tool here.

The term "WebLogo" was already explained in the original text. To make the description clearer, we have now added the number of GP5 sequences used to generate the logo to the sentence in lines 162–163. "The web logo displaying the frequency of amino acids at the N-terminus of 210 GP5 sequences." The tool itself is described in the Methods section.

Lines 214-216: the only reliable part of the prediction are the TMs

We agree that only parts of the ectodomain are reliably predicted and have revised the sentence (line 224) accordingly. It now reads: "Overall, the PAE analysis confirms that parts of the ectodomain and the transmembrane regions of the GP5/M dimer are predicted with reasonable structural reliability, while the endodomain predictions are of low quality."

Lines 225-227: the depth of the multiple sequence alignment used by AlphaFold should give clues about the prediction quality.

We examined the depth of the MSAs generated by AlphaFold for both EAV and PRRSV and have now incorporated this information into the Results section. The revised text (lines 256-262) reads: "This difference likely stems from the greater availability of PRRSV sequences in the GenBank database, **over 1,000 full-length GP5 sequences and roughly 100 M sequences contributed to the MSA**, providing a richer dataset for identifying covarying amino acid pairs during evolution—a critical factor for accurate structure prediction in AlphaFold. In contrast, **the EAV dataset is both smaller and often incomplete, approximately 100 M and 450 GP5 sequences in total, with only about 250 GP5 sequences covering the central region,** constrain the reliability of the EAV model, especially in the endodomain.

Line 245: Can we really say it is high confidence if the pLDDT values are <70? This reviewer has some doubts.

We changed the sentence to the following: "The ectodomain of GP5 in equine arteritis virus (EAV) is predicted with <u>moderate to</u> high confidence,"

Line 250: the proteins are homologous, allowing to compute a multiple sequence alignment, they do not "reveal limited homology" but reveal few conserved residues and an insertion in EAV GP5.

We changed the sentence to the following: "Multiple sequence alignments of the EAV GP5 ectodomain with PRRSV-1 (Lelystad) and PRRSV-2 (VR-2332) reveal <u>an insertion in EAV GP5 and few conserved residues</u> ~~limited homology~~ only in the N-terminal region,

Line 269-273: a detailed description of the moderate confidence predicted EAV GP5 ectodomain is not really necessary.

We thank the reviewer for this comment. We agree that the predicted ectodomain is of only moderate confidence. However, we believe that a brief description of the model shown in Fig. 2b is still important, as it provides the structural context necessary for interpreting the subsequent epitope analysis.

Line 283: Single residue epitopes? I don't think they exist… How were these 'epitopes' identified? How were the antibodies generated?

We agree that the original wording was misleading, as monoclonal antibodies do not recognize single amino acids in isolation. The cited studies mapped escape mutations to specific residues, which served as centers of antibody-sensitive regions rather than true single-residue epitopes. To avoid confusion, we have revised the text (lines 329-332) as follows: "Initial analyses of viral

mutants that escape neutralisation by monoclonal antibodies from mice identified four discrete epitopes within the GP5 ectodomain (35, 36). Two of these, designated sites A and B, correspond to epitopes centred on amino acids 31 and 43, respectively. The remaining sites, C and D, are broader regions spanning residues 49–72 and 80–88, respectively.

Line 315ff: How many sequences were available for these predictions and how do they represent the variability of EAV field strains? Please state here in the text.
For the ConSurf analysis, 250 M-protein sequences and 208 GP5 sequences were retrieved from the UniRef90 database, which provides broad antigenic representation while minimizing redundancy. This information has now been added to the main text (line 365) and is also described in the Methods section.

Line 316: WebLogo is more accurately described in legend of Figure 4
WebLogo is indeed described in more detail in the legend of Figure 4 and also in the methods section, and for this reason it is only briefly mentioned in the main text.

Line 352f: What is the likelihood that an amino acid exchange from isoleucine to valine will cause significant biological property changes of the protein?
We agree that the likelihood is low. To clarify this point, we have revised the sentence (line 403) to read: "Residue 55 is variable, typically occupied by isoleucine or valine, which represent a conservative substitution, and is followed by invariant residues Ile56 and Thr57.

Figure 5C-E: please show the predictions in the context of the full protein. Are they compatible with the overall structure of the heterodimer?
Based on its own statements and publications by others, Alphafold should not be used to evaluate the effects of replacing individual amino acids on the structure of an entire protein. This information is now included in the text (lines 437-440):
„Since Alphafold should not be used to assess the effect of single amino acids exchanges on the structure of an entire protein (55) ~~Structural~~ we predicted ~~ions of~~ only the region encompassing helices 3 and 4 ~~were performed~~ using wild-type and mutant sequences."

Line 438ff: How can you be sure that this is structural cohesion and not a prediction bias?
We agree that structural predictions can introduce bias. Our statement is therefore not intended to imply experimentally validated structural cohesion but rather to highlight that conserved prolines and acylation sites are expected, based on known biophysical effects, to promote stabilizing interactions between the TM regions. We have adjusted the wording accordingly (lines 495-496).

"Together, these conserved motifs are consistent with stabilizing interactions within the GP5/M transmembrane region of arteriviruses."

Paragraph on structural homology with coronavirus proteins: Please include structure overlays as a figure to visualise your findings.

We performed the requested structural overlay analysis and added the result to S11 Fig. The corresponding description has been added to the main text (lines 504–511).

"Attempts to align the GP5/M transmembrane regions with those of the long or short M isoforms did not produce meaningful structural matches, while alignment with ORF3a resulted in an RMSD of approximately 8 Å. This mismatch is attributable to distinct interhelical angles that alter the overall topology of the transmembrane bundle.

Line 452: the highest sequence conservation

Corrected as suggested

Line 454: How was the homology relationship between Orf3 and GP5/M demonstrated ?

We exchanged "homology" by "sequence similarity".

Line 475: if there was high sequence identity we could say close homologs. In the present case we could have (very) remote homologs, only if the homology relationship has been demonstrated.

We assume the referee refers to line 465 in the original manuscript, now line 526: "Taken together, the relatively high sequence homology and conserved structural features among the GP5/M heterodimer, M homodimer, and Orf3a homodimer suggest a deep evolutionary relationship across members of the Nidovirales order." To address the point of the referee we exchanged "homology" by "conservation" in this sentence.

Line 506ff: Given the high sequence variability of epitopes B and C, how can they be useful for vaccine antigen design? Will a mosaic design really be sufficient? And how should it be administered?

We agree that sequence variability is a challenge; however, our model suggests that the underlying β-sheet scaffold remains structurally conserved, providing a stable basis for antigen design. We have updated the Discussion to clarify how consensus or polyvalent approaches, supported by modern delivery systems like DC-targeting and viral vectors, can achieve the necessary antigenic breadth to overcome this drift. The revised text (lines 569-583) is as follows:

"Given the high surface accessibility and spatial proximity of epitopes B and C at the apex of the GP5 ectodomain, recombinant proteins representing this region may serve as promising

vaccine candidates. Although these epitopes are characterized by high sequence variability, our model indicates that they are supported by the β-sheet structure stabilized by disulfide linkages. This structural stability might preserve the native conformation of the epitope and enhance the likelihood of eliciting neutralizing antibodies. To broaden protective efficacy, the recombinant protein should be engineered to represent the consensus sequence across EAV lineages or incorporate a polyvalent mixture of amino acids at each position that reflect the diversity of epitopes found in currently circulating EAV strains. This design is expected to induce a cross-reactive antibody response capable of neutralizing prevalent viral variants. Moreover, fusing this region to a dendritic cell-targeting peptide could facilitate more robust antibody responses. For practical application, such a vaccine could be administered via intramuscular injection using modern adjuvants or through viral vector delivery to ensure both robust mucosal protection and systemic immunity."

Line 517ff: Couldn't this also imply that these sites need to be more conserved due to functional constraints? Isn't it in the interest of the virus to have functionally important epitopes being less dominant? What about broadly neutralising epitopes?

We thank the reviewer for this thoughtful comment. By thinking about this point, we found the overlapping reading frames of GP5 and ORF5a as a possible explanation for conservation at these sites. More precisely the codon for residue 31 in GP5 coincides with the ORF5a stop. The revised passage (lines 589-598) reads:

"The genomic regions encoding GP5 and the ORF5a protein overlap, placing helix 1 and part of helix 2 within this shared reading frame. Specifically, the codon for residue 31 in GP5 coincides with the ORF5a stop codon. Consequently, most nucleotide substitutions at this position would result in a read-through of the ORF5a stop codon, leading to an elongated and likely non-functional ORF5a protein. Given that ORF5a is beneficial for EAV infectivity, this dual-coding requirement imposes a severe evolutionary constraint that likely accounts for the observed invariance of site A. These invariant regions, alongside the highly conserved M protein, may therefore represent potential targets for broadly neutralizing antibodies, offering a pathway toward cross-strain protection that is less susceptible to the rapid antigenic drift observed at the molecular apex."
