# Supplementary material for: Computational modelling of the equine arteritis virus GP5/M Dimer: Implications for immune evasion and virulence
Source: PLoS One. 2026 Mar 10;21(3):e0344287. doi: 10.1371/journal.pone.0344287 (PMC12974795; doi:10.1371/journal.pone.0344287)
Supplement: Attachment — Submitted filename: Rev_MAH_01012026.docx [file pone.0344287.s013.docx]

**General Comments to the Editor**

The manuscript presents an AlphaFold3-based structural model of the Equine Arteritis Virus (EAV) GP5/M heterodimer, supported by confidence metrics and comparative analyses with PRRSV. The authors map antibody epitopes, analyze sequence variability, and discuss potential mechanisms of immune evasion and virulence. The computational work is technically sound and the analyses are thorough.

However, several conclusions—particularly those related to virus budding—are **overstated** given the absence of experimental validation. Structural similarity to PRRSV or SARS-CoV-2 proteins alone is insufficient to establish a mechanistic role in budding. These claims should be framed more cautiously as hypotheses derived from predictive modeling. Overall, the study offers useful structural and evolutionary insights but would benefit from moderated claims and clearer separation between prediction and experimentally supported function.

**Comments to the Authors**

**Title**: The title implies experimentally validated mechanisms (“structural basis for virus budding”) that are not demonstrated. A more conservative title reflecting the predictive nature of the study is recommended.

**Introduction**: While the study focuses on EAV, the Introduction places disproportionate emphasis on PRRSV GP5/M biology. This weakens the narrative focus and makes EAV appear primarily as a comparative extension. The Introduction should more clearly define the specific knowledge gap for EAV and justify the comparative framework.

**Interpretation of Structural and Evolutionary Comparisons**: The reported structural similarities between arterivirus GP5/M and SARS-CoV-2 M/ORF3a are interesting, but conclusions should be limited to conserved topology and fold. Functional or evolutionary equivalence cannot be inferred from AlphaFold3 predictions alone, particularly given their static nature.

**Limitations**: The study relies exclusively on computational modeling. Regions with low confidence, especially the endodomains, limit interpretation of cytoplasmic interactions. Claims related to virus budding and assembly should therefore be explicitly presented as speculative and hypothesis-generating.

**Conclusion**: Despite these limitations, the manuscript provides a detailed structural model and contributes novel insights into GP5/M organization, epitope distribution, and sequence variability. With a revised title, a more EAV-focused Introduction, and tempered functional claims, the study would meet PLOS ONE standards and serve as a useful resource for the field.

Top of FormBottom of Form
